# Number of negative lymph nodes is associated with disease-free survival in patients with breast cancer

**DOI:** 10.1186/s12885-015-1061-z

**Published:** 2015-02-07

**Authors:** San-Gang Wu, Jia-Yuan Sun, Juan Zhou, Feng-Yan Li, Qin Lin, Huan-Xin Lin, Xun-Xing Guan, Zhen-Yu He

**Affiliations:** 1 Xiamen Cancer Center, Department of Radiation Oncology, the First Affiliated Hospital of Xiamen University, Xiamen, 361003 People’s Republic of China; 2 Department of Radiation Oncology, Sun Yat-sen University Cancer Center, State Key Laboratory of Oncology in South China, Collaborative Innovation Center of Cancer Medicine, Guangzhou, 510060 People’s Republic of China; 3 Xiamen Cancer Center, Department of Obstetrics and Gynecology, the First Affiliated Hospital of Xiamen University, Xiamen, 361003 People’s Republic of China

**Keywords:** Breast cancer, Mastectomy, Negative lymph nodes, Prognosis, Disease-free survival

## Abstract

**Background:**

The aim of this study was to evaluate the prognostic value of the number of negative lymph nodes (NLNs) in breast cancer patients after mastectomy.

**Methods:**

2,455 breast cancer patients who received a mastectomy between January 1998 and December 2007 were retrospectively reviewed. The prognostic impact of the number of NLNs with respect to disease-free survival (DFS) was analyzed.

**Results:**

The median follow-up time was 62.0 months, and the 5-year and 10-year DFS was 87.1% and 74.3%, respectively. The DFS of patients with >10 NLNs was significantly higher than that of patents with ≤10 NLNs, and the 5-year DFS rates were 87.5% and 69.5%, respectively (*P* < 0.001). Univariate Cox analysis showed that the NLN count (continuous variable) was a prognostic factor of DFS (hazard ratio [HR] = 0.913, 95% confidence interval [CI]: 0.896-0.930, *P* < 0.001). In multivariate Cox analysis, patients with a higher number of NLNs had a better DFS (HR = 0.977, 95% CI: 0.958-0.997, *P* = 0.022). Subgroup analysis showed that the NLN count had a prognostic value in patients at different pT stages and pN positive patients (log-rank *P* < 0.001). However, it had no prognostic value in pN0 patients (log-rank *P* = 0.684).

**Conclusions:**

The number of NLNs is an independent prognostic factor of DFS in breast cancer patients after mastectomy, and patients with a higher number of NLNs have a better DFS.

## Background

Though the survival rates of patients receiving sentinel lymph node biopsy and of patients receiving axillary lymph node dissection are similar in a certain specific populations with breast cancer [1,2], and sentinel lymph node biopsy can decrease postoperative arm lymphedema [3,4], the axillary lymph node status is still one of the most important prognostic indicators of breast cancer patients and is useful for guiding treatment. Moreover, it is also important in the Union for International Cancer Control/American Joint Committee on Cancer (UICC/AJCC) tumor, node, metastasis (TNM) staging system for breast cancer.

Axillary lymph node dissection is an important method for determining the axillary lymph node status in breast cancer patients. In theory, the survival of breast cancer patients is improved by removing more axillary lymph nodes. However, the prognostic value of the number of axillary lymph nodes removed is controversial [5-7]. Because both positive and negative lymph nodes are removed, it is difficult to accurately determine the proper number of lymph nodes that should be removed.

The number of negative lymph nodes (NLNs) removed is obtained by subtracting the number of positive lymph nodes from the total number of removed lymph nodes. Because removing more NLNs may reduce the possibility of occult lesions and thus improve the prognosis, the number of NLNs removed may be an indicator of the degree of the appropriateness of axillary lymph node dissection. The prognostic value of the number of NLNs removed in esophageal cancer, colorectal cancer, and cervical cancer has been proven [8-11]. However, there have been few studies on its prognostic value of the number of NLNs removed for breast cancer [12,13]. The purpose of this study was to determine the prognostic value of the number of NLNs with respect to disease-free survival (DFS) of breast cancer patients after mastectomy.

## Methods

### Patients

The records of breast cancer patients treated at Sun Yat-Sen University Cancer Center between January 1998 and December 2007 were retrospectively reviewed. The inclusion criteria were: 1) Females who had histologically confirmed unilateral invasive breast cancer; 2) Underwent mastectomy together with axillary lymph node dissection and the number of removed axillary lymph nodes was more than 10; 3) The tumor was completely removed and the margins were negative; 4) No neoadjuvant chemotherapy was administered before surgery and postoperative treatments including chemotherapy, radiotherapy, and endocrine therapy were performed based on the tumor stage and hormone receptor status. The study was approved by the ethics committee of Sun Yat-Sen University Cancer Center. All patients provided written consent for storage of their information in the hospital database, and for use of this information for research purposes.

### Clinicopathologic factors and lymph node status

The risk of recurrence was evaluated according to the clinicopathological characteristics and immunohistochemical factors which included age, menopause status, pT stage, pN stage, and estrogen receptor (ER), progesterone receptor (PR), and human epithelial growth factor receptor family 2 (Her2) status. ER and PR positive was defined as more than 1% positive cells on immunohistochemical analysis. Her2-positivity was defined as a 3+ immunohistochemical result or a 2+ immunohistochemical result confirmed by fluorescent in situ hybridization (FISH). pT stage and pN stage were consistent with the UICC/AJCC TNM classification (7^th^ Edition), and pN stages were defined as follows: pN0, no regional lymph node metastasis identified histologically; pN1, metastasis in 1–3 lymph nodes; pN2, metastasis in 4–9 lymph nodes; pN3, metastasis in ≥10 lymph nodes. The number of removed NLNs was defined as the number of positive lymph nodes subtracted from the total number of removed lymph nodes.

### Histopathological examination of resected lymph nodes

All resected specimens were submitted for pathologic examination. Pathologists examined all slides to evaluate the depth of the primary tumors and node involvement, which were separately labeled by the surgeons in a routine manner. One section from each lymph node was analyzed after hematoxylin and eosin (H&E) staining. Lymph nodes that were examined included those that were embedded in the en bloc specimen and not labeled by surgeons, but were identified by the pathologists. The lymph node number was counted on low-power field microscopy. The total number of resected lymph nodes was the sum of the lymph nodes removed form the axilla. The number of metastatic lymph nodes, and the number of removed nodes was determined.

### Follow-up and survival endpoints

Follow up was performed 3–6 months after surgery by hospital visit, telephone, or mail correspondence. Because all patients in the present study received adjuvant treatment according to the stage and hormone receptor status, the endpoint was DFS. For patients with recurrence, survival time was determined from the date of surgery to the date of locoregional recurrence and/or distant metastasis.

### Statistical analysis

The *χ*^2^ and Fisher’s exact tests were used to analyze the differences between qualitative data. Recognizing that the total number of NLNs removed may be subjected to incomplete counting or natural interindividual variation in nodal distribution, the variable was examined as a categorical variable based on quartiles. Calculation of survival rates were plotted by the Kaplan-Meier method, and compared using the log-rank test. Univariate and multivariate Cox regression model analyses were performed. All analyses were performed using the SPSS statistical software package version 16.0 (IBM Corporation, Armonk, NY, USA). A value of *P* < 0.05 was considered statistically significant.

## Results

### Clinicopathological characteristics and lymph node dissection data

A total of 2,455 patients were included in the analysis, and the clinical features are shown in Table 1. The median number of removed lymph nodes was 15 (25^th^ percentile = 12, 75^th^ percentile = 18; range, 10–73), 1,263 patients were node negative (51.4%), and 1,192 patients were node positive (48.6%). Of the patients, 769, 207 and 216 were pN1, pN2 and pN3, respectively.

**Table 1:** **Correlation between the number of negative lymph nodes removed and clinicopathologic factors**

Characteristic		Number of negative lymph nodes				*P*value
		0-10 (n = 607)	11-13 (n = 706)	14-16 (n = 554)	17-40 (n = 588)	
Age						
<35	234	68 (11.2)	51 (7.2)	51 (9.2)	64 (10.9)	0.054
≥35	2221	539 (88.8)	655 (92.8)	503 (90.8)	524 (89.1)	
Menopause						
Premenopause	1641	402 (66.2)	460 (65.2)	354 (63.9)	425 (72.3)	0.012^*^
Postmenopause	814	205 (33.8)	246 (34.8)	200 (36.1)	163 (27.7)	
pT stage						
T1	812	159 (26.2)	259 (36.7)	190 (34.3)	204 (34.7)	<0.001^*^
T2	1428	352 (58.0)	398 (53.4)	323 (58.3)	355 (60.4)	
T3	155	71 (11.7)	41 (5.8)	25 (4.5)	18 (3.1)	
T4	60	25 (4.1)	8 (1.1)	16 (2.9)	11 (1.8)	
pN stage						
N0	1263	120 (19.8)	430 (60.9)	342 (61.7)	371 (63.1)	<0.001^*^
N1	769	185 (30.5)	220 (31.2)	174 (31.4)	190 (32.3)	
N2	207	115 (18.9)	44 (6.2)	30 (5.4)	18 (3.1)	
N3	216	187 (30.8)	12 (1.7)	8 (1.5)	9 (1.5)	
ER						
Negative	953	245 (40.4)	252 (35.7)	219 (39.5)	237 (40.3)	0.304
Positive	1292	321 (52.9)	384 (54.3)	287 (51.8)	300 (51.0)	
Unknown	210	41 (6.7)	70 (10.0)	48 (8.7)	51 (8.7)	
PR						
Negative	824	207 (34.1)	223 (31.5)	198 (35.7)	196 (33.3)	0.398
Positive	1421	359 (59.1)	413 (58.5)	308 (55.6)	341 (58.0)	
Unknown	210	41 (6.8)	70 (10.0)	48 (8.7)	51 (8.7)	
Her-2						
Negative	1377	329 (54.2)	395 (56.0)	307 (55.4)	346 (58.8)	0.460
Positive	666	168 (27.7)	183 (25.9)	157 (28.3)	158 (26.9)	
Unknown	412	110 (18.1)	128 (18.1)	90 (16.3)	84 (14.3)	

Data are presented as number (percentage).

ER, estrogen receptor; PR, progesterone receptor; Her-2, human epidermal growth factor receptor-2.

^*^P < 0.05 indicates a significant difference.

The median number of NLNs removed was 13 (25th percentile = 11, 75th percentile = 16; range, 0–40). Examination of the number of NLNs removed as a categorical variable based on quartiles showed the number of patients with 0–10 NLNs removed (group 1) was 607, the number with 11–13 NLNs removed (group 2) was 706, the number with 14–16 NLNs removed (group 3) was 554, and the number with 17–40 NLNs removed (group 4) was 588.

The NLN count was associated with menopause status, pT stage, and pN stage (*P* < 0.05). The NLN count was not associated with age or ER, PR, and Her-2 status (all, *P* > 0.05) (Table 1).

### Relationship between the number of NLNs removed and DFS

**Figure 1:**
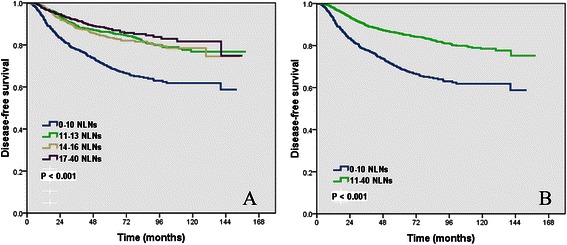
**Impact of the number of negative lymph nodes on disease-free survival for 4 categories of patients (A), and patients with group 1 vs. group 2-4 (B).**

The median follow-up time was 62.0 months (range, 6 to 158 months), and 477 patients had local recurrence and/or distant metastasis. The 5- and 10-year DFS rates were 81.7% and 74.3%, respectively. The number of NLNs removed had a significant impact on DFS, the 5-year DFS in group 1, group 2, group 3, and group 4 were 69.5%, 86.0%, 83.3%, and 87.6%, respectively. The 10-year DFS in group 1, group 2, group 3, and group 4 were 61.8%, 76.8%, 78.5%, and 81.7%, respectively (*P* < 0.001) (Figure 1A). However, intersection and overlapping were observed between the survival curves of patients in group 2, 3 and 4; therefore, we combined these 3 groups. The analysis with these groups combined showed that the 5- and 10-year DFS rates were 85.7% and 78.5% in patients with > 10 NLNs removed, which were significantly higher than 69.5% and 61.8% of patients with ≤ 10 NLNs removed (*P* < 0.001) (Figure 1B).

### Analysis of prognostic factors

Univariate analysis showed that age, pT stage, pN stage, ER status, PR status, and the number of NLNs removed (continuous variables) were prognostic factors affecting DFS (all, *P* < 0.001) (Table 2).

**Table 2:** **Univariate and multivariate analyses of disease-free survival**

Characteristic	Univariate			Multivariate		
	HR	95% CI	*P*value	HR	95% CI	*P*value
Age						
<35	1			1		
≥35	0.618	0.476-0.803	<0.001^*^	0.679	0.522-0.883	0.004^*^
Menopause						
Premenopause	1			—		
Postmenopause	0.995	0.822-1.205	0.960	—		
pT stage						
T1	1			1		
T2	1.699	1.361-2.120	<0.001^*^	1.361	1.088-1.704	0.007^*^
T3	2.844	2.036-3.972	<0.001^*^	1.585	1.116-2.252	0.010^*^
T4	3.150	1.933-5.134	<0.001^*^	1.798	1.091-2.963	0.021^*^
pN stage						
N0	1			1		
N1	2.187	1.746-2.740	<0.001^*^	2.083	1.658-2.616	<0.001^*^
N2	3.139	2.322-4.244	<0.001^*^	2.506	1.817-3.456	<0.001^*^
N3	6.725	5.236-8.638	<0.001^*^	4.575	3.315-6.313	<0.001^*^
ER						
Negative	1			1		
Positive	0.586	0.504-0.682	<0.001^*^	0.720	0.582-0.892	0.003^*^
PR						
Negative	1			1		
Positive	0.620	0.533-0.722	<0.001^*^	0.790	0.636-0.980	0.032^*^
Her2						
Negative	1			—		
Positive	0.977	0.871-1.096	0.693	—		
Number of NLNs (continuous variable)	0.913	0.896-0.930	<0.001^*^	0.977	0.958-0.997	0.022^*^

DFS, disease-free survival; HR, hazard ratio; CI, confidence interval; ER estrogen receptor; PR, progesterone receptor; Her-2, human epidermal growth factor receptor-2; NLNs, negative lymph nodes.

^*^P < 0.05 indicates a significant difference.

Multivariate analysis showed that the number of NLNs removed was an independent prognostic factor of DFS; patients with a higher number of NLNs had a better DFS (hazard ratio [HR] = 0.977, 95% confidence interval [CI]: 0.958-0.997, *P* = 0.022). In addition, age, pT stage, pN stage, ER status, and PR status were also independent risk factors of DFS (all, *P* < 0.05) (Table 2).

### Impact of the number of NLNs removed on DFS by pT stage

**Figure 2:**
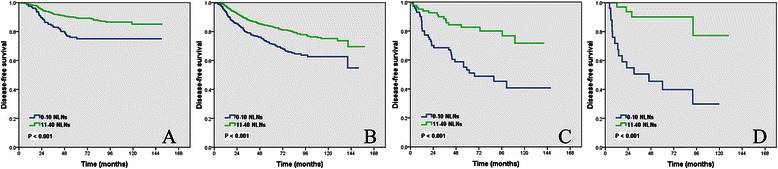
**Impact of the number of negative lymph nodes on the disease-free survival of pT1 (A), pT2 (B), pT3 (C), and pT4 (D) patients.**

Subgroup analysis of the impact of the number of NLNs removed on DFS by different pT stage showed that patients with a higher number of NLNs removed at all pT stages had better DFS (log-rank *P* < 0.001 for pT1, *P* < 0.001 for pT2, *P* < 0.001 for pT3, and *P* < 0.001 for pT4) (Figure 2A-D).

### Impact of the number of NLNs removed on DFS by pN stage

**Figure 3:**
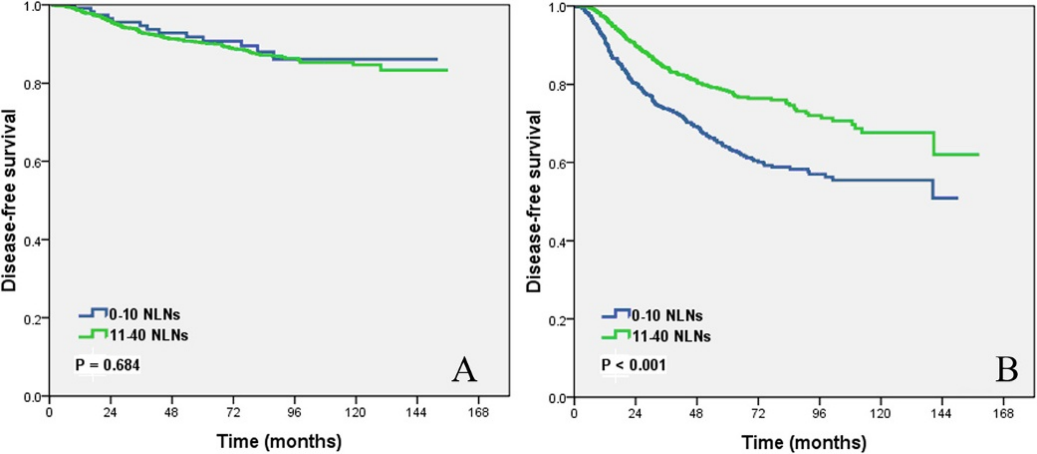
**Impact of the number of negative lymph nodes on the disease-free survival of pN0 patients (A) and pN positive patients (B).**

Subgroup analysis of the impact of the number of NLNs removed on DFS by different pN stage showed that the NLN count removed had no impact on DFS in pN0 patients (log-rank *P* = 0.684). However, a higher number of NLNs removed indicated better DFS in pN-positive patients (log-rank *P* < 0.001) (Figure 3A,B).

## Discussion

In the present study, we investigated the impact of the number of NLNs removed after mastectomy in breast cancer patients and found that the number of NLNs removed was an independent prognostic factor of DFS. Patients with a higher number of NLNs removed had better DFS, and the number of NLNs had a prognostic value in patients with different pT stages and in pN-positive patients.

Because lymph node dissection includes positive lymph nodes, it is difficult to accurately estimate the appropriate number of lymph nodes to remove. Our results are consistent with those of other studies which have examined the prognostic value of the number of NLNs removed in breast cancer patients [12,13]. Karlsson et al. found that the number of NLNs removed was an independent factor affecting prognosis; patients with ≥ 10 NLNs removed had a better prognoses than patients with <10 NLNs removed, which affected node positive patients but not node negative patients [12]. Kuru reported that patients with >15 NLNs removed had a better prognoses than those with fewer removed [13]. The mechanism underlying why the number of NLNs can be used to predict the survival of breast cancer patients is unclear. Insufficient lymph node dissection may result in inaccurate lymph node staging, and removing more lymph nodes makes for more accurate determination of the lymph node status. Therefore, studies have proposed the “stage migration” hypothesis that obtaining accurate information of lymph nodes and determining the lymph node stage by removing more lymph nodes to decrease the probability of error of nodal stage. Schaapveld et al. found that removing more lymph nodes resulted in a better survival, which supports the “stage migration” hypothesis [14].

At present, H&E staining is a commonly used method for detecting positive lymph nodes. However, immunohistochemical methods can reveal occult lesions in lymph nodes [15,16]. It is obvious that an increased number of NLNs removed increases the potential for the identification of micrometastases. Because immunohistochemical methods were not used for examination of the lymph nodes in the present study, removing a higher number of NLNs might eliminate some potential remnant lesions, and this can explain the fact that patients with a higher number of NLNs removed had better DFS in the present study, which also supports the “stage migration” hypothesis. Moreover, it should be mentioned that the number of NLNs removed may be related to the host immune response against tumor cells, and the molecular biology of tumor cells [17,18]. A study examining colorectal cancers showed there was a significant correlation between lymphocyte response against tumor cells and patients survival [19]. Hence, it is meaningful to further investigate the number of NLNs removed and the lymphocyte response against tumor cells for the individualized treatment of breast cancer.

The American College of Surgeons Oncology Group (ACOSOG) Z0011 trial showed that the local recurrence and survival rates of patients who underwent breast-conserving surgery with negative sentinel lymph node or 1–2 positive sentinel lymph nodes were not affected by whether axillary lymph node dissection was performed or not, and thereby suggests that axillary lymph node dissection should not be carried out for these patients [1,2]. However, the report of the St-Gallen International Breast Cancer Conference in 2013 indicates that axillary lymph node dissection should be performed for patients who cannot receive radiotherapy or who have ≥ 3 metastatic sentinel lymph nodes [20]. Z0011 trial participants received breast conserving surgery and radiotherapy, patients enrolled in the present study were treated with mastectomy and irradiation was performed based on the tumor stage and lymph node status.

In the present study, subgroup analysis showed that the number of NLNs removed only affected the DFS of node positive patients. Though it was reported that the number of removed lymph nodes might affect the local recurrence rate of node positive patients [21], we found that the number of NLNs removed did not affect the DFS of pN0 patients and thus consider that sentinel lymph node biopsy is sufficient for the determination of lymph node status in node-negative patients.

There are limitations of the present study that must be considered. First, the study was a single center retrospective study, and thus may not represent the majority of the population. However, the number of cases was large. Second, the optimal number of NLNs removed is not consistent with that of other studies. This may be related to differences in clinical data and surgical procedures, and prospective multicenter studies should be carried out to identify an exact value and a more appropriate cut-off number of the NLNs that should be removed.

## Conclusions

In conclusion, our study indicates that the number of NLNs removed is an important factor affecting the DFS of breast cancer patients after mastectomy, and patients with a higher number of NLNs removed have a better prognosis. However, our result should be verified by further studies, and the related mechanism should be studied to provide a choice for the postoperative treatment of breast cancer.

## Abbreviations

UICC/AJCC: Union for International cancer control/american joint committee on cancer
TNM: Tumor, node, metastasis
NLNs: Negative lymph nodes
DFS: Disease-free survival
ER: Estrogen receptor
PR: Progesterone receptor
Her2: Human epithelial growth factor receptor family 2
FISH: Fluorescent in situ hybridization
H&E: Hematoxylin and eosin
HR: Hazard ratio
CI: Confidence interval
ACOSOG: American College of Surgeons Oncology Group
